# Epicardial adipose tissue volume and nephropathy in people living with type 2 diabetes: the Kidn-EAT study

**DOI:** 10.1186/s12933-026-03311-z

**Published:** 2026-07-30

**Authors:** Anaïs Andrieu, Sopio Tatulashvili, Siwar Hammami, Mohamed Lamine Mariko, Imen Rezgani, Lucie Allard, Meriem Sal, Ines Barka, Mohamed Zerguine, Sébastien Homs, Pierre-Yves Brillet, Emmanuelle Fabre, Coralie Bloch-Queyrat, Hélène Bihan, Emmanuel Cosson

**Affiliations:** 1 Department of Endocrinology-Diabetology-Nutrition, Avicenne Hospital, AP-HP, CRNH-IdF, CSO Ile de France Nord, Sorbonne Paris Nord University, Sorbonne Paris Cité, 125 rue de Stalingrad, Bobigny, France; 2 INSERM, INRAE, CNAM, Center of Research in Epidemiology and StatisticS (CRESS), Nutritional Epidemiology Research Team (EREN), Université Sorbonne Paris Nord and Université Paris Cité, 93017 Bobigny, France; 3 Clinical Research Unit, AP-HP, Avicenne Hospital, Bobigny, France; 4 Department of Radiology, AP-HP, Avicenne Hospital, Bobigny, France; 5 INSERM 1272, Sorbonne Paris Nord Paris University, 93009 Bobigny Cedex, France; 6 Biochemistry Department, Avicenne and Jean Verdier Hospitals, AP-HP, Sorbonne Paris Nord University, Bobigny, France; 7 UMR INSERM U1349 “Signalisation, Microenvironnement et Hémopathies Lymphoïdes, Sorbonne Paris Nord University, 93000 Bobigny, France; 8 Laboratoire Educations et Promotion de la Santé UR 3412, UFR Santé, Médecine, Biologie Humaine, Université Paris Sorbonne Paris Nord, 74, Rue Marcel Cachin, 93017 Bobigny Cedex, France

**Keywords:** Epicardial adipose tissue, Nephropathy, Albuminuria/creatininuria ratio, Estimated glomerular filtration rate, Type 2 diabetes, Visceral adipose tissue

## Abstract

**Introduction:**

Epicardial adipose tissue (EAT), a visceral adipose tissue located between the pericardium and the myocardium, has been linked to cardiac issues. The present study aimed to evaluate the association between EAT volume and the presence of nephropathy and its components, i.e. albuminuria/creatininuria ratio (ACR) and estimated glomerular filtration rate (eGFR) classes, in patients living with type 2 diabetes (PwT2D).

**Materials and methods:**

The present study is a cross-sectional analysis of a retrospective cohort of 700 PwT2D who had a computed tomography scan to measure both their coronary artery calcium score and EAT volume (proprietary prototype, GE HealthCare), and available data for both eGFR and ACR.

**Results:**

A total of 700 PwT2D (332 women) were included, of whom 295 patients had nephropathy (42.1%), including 87 (12.4%) with eGFR < 60 ml/min/1.73m^2^ and 258 (36.9%) with ACR ≥ 3 mg/mmol. Mean age (± standard deviation) was 60.3 ± 10.4 years old and 44.8% had obesity. EAT volume was higher in patients with nephropathy compared to those without (respectively, 103.6 ± 39.8 versus 93.8 ± 36.8 cm^3^, p = 0.001). EAT volume differed across eGFR classes (≥ 60 versus < 60 mL/min/1.73m^2^): respectively 96.6 ± 38.1 and 107.7 ± 39.2 (p = 0.012) and ACR classes (< 3; between 3 and 29.9; ≥ 30 mg/mmol): respectively 94.7 ± 36.9, 106.0 ± 40.2, and 95.7 ± 39.8 cm^3^ (p = 0.004). EAT volume was weakly correlated to eGFR (r = -0.146; p < 0.001) and to ACR (r = 0.124; p = 0.001). After adjustment for major confounding factors, each 10 cm^3^ increase of EAT volume was associated with a 5.8% increase in the odds of nephropathy (OR = 1.058 [95% CI 1.007–1.112], p = 0.026) and a 5.9% increase in the odds of ACR ≥ 3 mg/mmol (OR = 1.059 [95% CI 1.008–1.114], p = 0.024), but not with eGFR < 60 mL/min/1.73m^2^ (OR = 1.047 [95% CI 0.975–1.123], p = 0.204).

**Conclusion:**

EAT volume was associated with nephropathy and especially with increased ACR in PwT2D. This association may be partly explained by metabolic and inflammatory mechanisms, potentially driven by EAT and/or other associated ectopic fat depots in PwT2D.

**Graphical abstract:**

**Figure:**
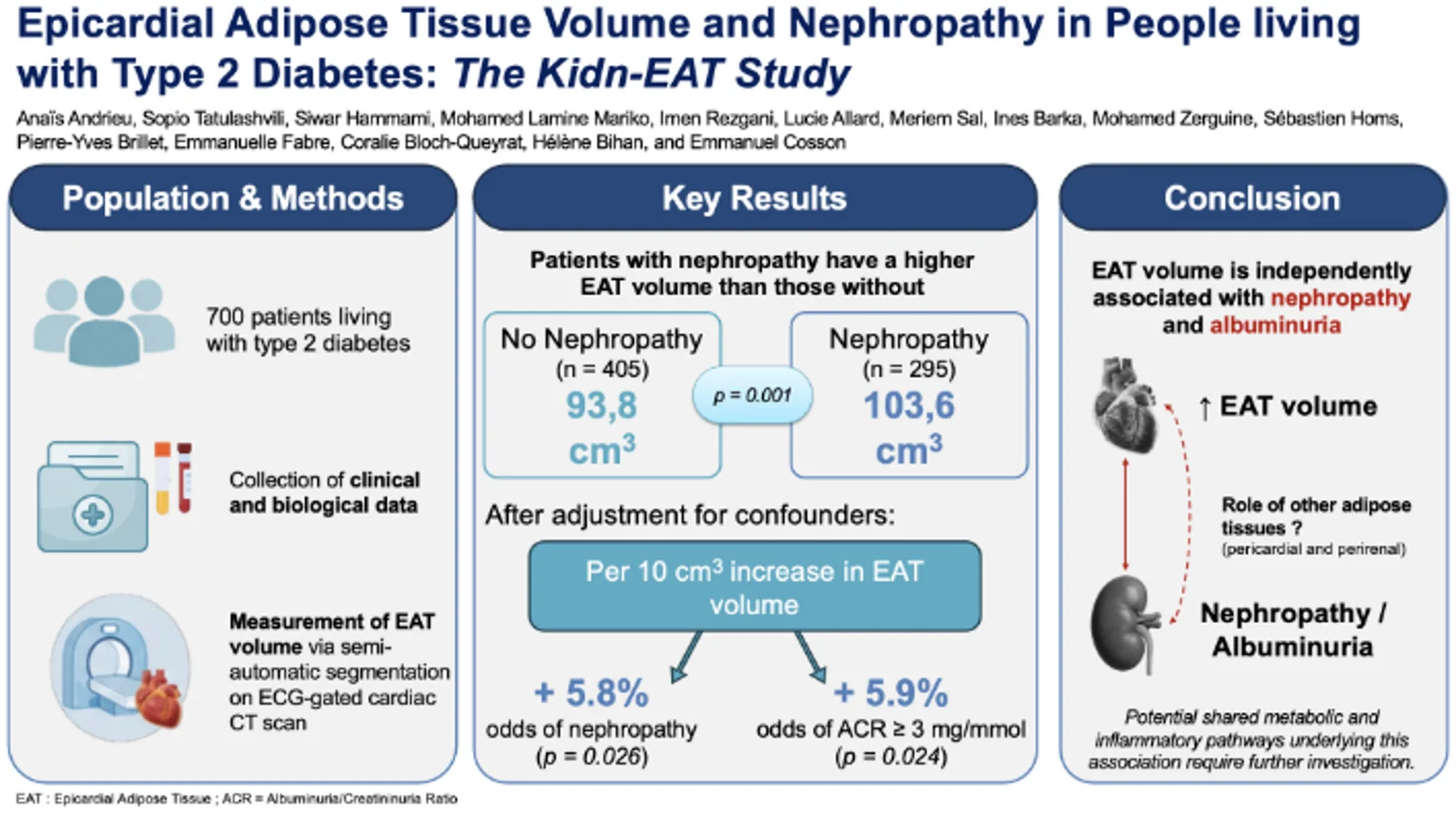

**Supplementary Information:**

The online version contains supplementary material available at https://doi.org/10.1186/s12933-026-03311-z.

## Introduction

Diabetes is one of the most common non-communicable diseases in the world, with an increasing prevalence of all types combined [1]. Nephropathy, which affects 30 to 40% of patients living with diabetes [2, 3], is currently the leading cause of end-stage renal failure worldwide as well as being an important cause of morbidity and mortality in this population [2–4]. It is defined by vasculo-glomerular damages leading to an increase in the albuminuria/creatininuria ratio (ACR) and subsequently a decrease in the estimated glomerular filtration rate (eGFR). Both ACR and eGFR are known to be markers of poor cardiovascular prognosis in patients living with diabetes, exacerbating morbidity and mortality [4].

Epicardial adipose tissue (EAT) has also been linked to cardiovascular risk in the general population [5, 6] as well as in patients living with diabetes [7–13]. Located between the pericardium and the myocardium, this tissue is considered a type of visceral adipose tissue. It is also metabolically active by synthesizing pro-inflammatory and pro-atherogenic markers, which may promote atherosclerosis [6, 14]. Interestingly, increased EAT volume in patients with chronic kidney disease is a better predictor of cardiovascular risk than abdominal visceral adipose tissue [15]. Specifically in patients living with diabetes, who show a higher risk of nephropathy, the EAT volume is known to be increased compared to healthy patients [16–19].

To date, only two studies were designed to specifically evaluate the association between EAT thickness —and not volume—and nephropathy in patients living with diabetes, only characterized by ACR [20, 21]. The first study showed in 200 patients living with type 2 diabetes an association between EAT thickness and ACR after adjusting for confounding factors that did not include hypertension [20]. The second study found a positive correlation between EAT thickness and ACR in 180 patients living with type 2 diabetes that disappeared after adjustment for body mass index (BMI) or hypertension [21]. The last two cohorts involved a limited number of patients, which limited the number of adjustment factors. Recently, the EPIDIAB study showed an association between fully automated EAT volume and nephropathy in a multicenter cohort of 1,253 patients with type 2 diabetes [9], but this association was not evident in the multivariable model. Although they defined nephropathy as eGFR < 60 ml/min/1.73m^2^ and/or ACR ≥ 3 mg/mmol in a large cohort, they did not assess the association of EAT volume with each component of nephropathy. Therefore, the association between EAT amount, particularly its volume, and diabetic nephropathy, and specifically both eGFR and ACR, warrants further investigation.

In this context, the present study aimed to assess the association between EAT volume and both ACR and eGFR in a sample large enough to allow adjustment for confounding factors. Our main hypothesis was that EAT volume would be higher in people with nephropathy than in those without. Identifying an association between EAT volume and nephropathy in patients with diabetes would reinforce the idea of EAT as an indicator of adverse metabolic risk in this patient group and could lead to new research avenues to improve our understanding of the nephroprotective effects of certain treatments.

## Methods

### Inclusion criteria

The present study used data collected from three observational studies involving individuals with diabetes who underwent a computed tomography (CT) scan to measure their coronary artery calcium (CAC) score for cardiovascular risk assessment [7, 8, 13, 22]. These studies included *(i)* consecutive asymptomatic individuals in primary coronary prevention who had a stress myocardial scintigraphy between 2009 and 2018, in the diabetes clinic at Jean Verdier Hospital, Bondy, France [8]; consecutive individuals admitted to the department of Diabetology-Endocrinology-Nutrition at Avicenne Hospital, Bobigny, France, who had (ii) a CAC score measured between 2019 and 2020 [7] and (iii) their arterial stiffness evaluated between 2018 and 2022 [13]. Note that Jean Verdier and Avicenne are sister hospitals and members of the Greater Paris University Hospital network.

Whether a patient had participated in more than one initial study, only one observation was considered in the dataset, named microEAT. MicroEAT included 1,093 individuals, of whom seven were excluded as they refused to participate in any subsequent observational study using this dataset. For the present study, named Kidn-EAT, we restricted the analysis to patients living with type 2 diabetes to ensure sufficient statistical power for adjustment for numerous confounders. As shown in the flowchart (Supplementary Fig. 1), we excluded 155 patients whose nephropathy status was unknown and 1 patient whose EAT volume could not be calculated. Accordingly, the final analysis was conducted on 700 patients. The Kidn-EAT study was validated by the local ethical committee on June 10, 2022 (CLEA-2022–266).

### Data collection

Data were extracted from patients’ medical records and collected anonymously in a secure health database: general data such as age, sex, ethnicity, current smoking status, diagnosed premature (before 55 years of age for men; before 65 years for women) coronary artery disease in first-degree relatives; medical history: diabetes duration and routine treatments before admission. Hypertension and dyslipidemia were self-reported and/or inferred from prescriptions for antihypertensive and lipid-lowering agents, respectively. Additionally, we collected data to measure possible overweight (BMI 25–29.9 kg/m^2^) and obesity (BMI ≥ 30 kg/m^2^). BMI was calculated using the formula: weight (kg) / height^2^ (m^2^). Weight and height were measured within 24 h of hospital admission. We collected information on retinopathy (detected by fundus photography or ophthalmoscopy), neuropathy (defined as any sign or symptom of polyneuropathy), and macroangiopathy (history of acute coronary syndrome, stroke, peripheral artery occlusive disease, 50% or greater stenosis measured by ultrasound examination).

The primary outcome was the presence of a nephropathy defined as eGFR < 60 ml/min/1.73m^2^ and/or ACR ≥ 3 mg/mmol. Patients were secondly classified in sub-groups according to their eGFR (≥ 60 and < 60 mL/min/1.73m^2^) and their ACR (< 3; 3–29.9 and ≥ 30 mg/mmol). eGFR was estimated using the chronic kidney disease-Epidemiology Collaboration equation based on serum creatinine measured by colorimetric enzymatic assay in one sample. ACR was measured on a single urine sample using immunoturbidimetry for albumin and colorimetric enzymatic assays for creatinine measurements.

Finally, we collected information on glycated hemoglobin (HbA1c) (HPLC dedicated assay using Tosoh HLC-723G11), total and HDL-cholesterol (colorimetric assay on homogenous phase and cholesterol dosage by cholesterol oxidase), triglycerides (colorimetric assay), and LDL-cholesterol (calculated using the Friedewald formula). C reactive protein (CRP) was also measured (immunoturbidimetry method). All these measurements were performed on serum from fasting individuals using a Cobas 6000 analyzer (Roche diagnostics, Meylan, France).

### Computed tomography (CT) imaging

EAT volume and CAC score were calculated using ECG-gated cardiac CT without contrast injection. All CT scans were performed with General Electrics (GE, Healthcare Digital, France) or Siemens (Healthineers, France) scanners. CAC score was calculated following manufacturer’s guidelines [23] using a dedicated tool available on Picture Archiving and Communication Systems platforms (either from Carestream Health, Rochester, NY or Philips Healthcare, Best, the Netherlands). In detail, the protocol used fixed parameters for all patients on both scanners: a slice thickness of 3 mm, an X-ray tube voltage of 120 kV and a field of view of 25 cm. The reconstruction kernel was 'soft' and 'mediastinal' in both cases, with the same generation of algorithm based on medium iterative reconstruction, ensuring very comparable image quality on both scanners.

EAT volume was measured with Volume Viewer software package (proprietary prototype, GE HealthCare) using a semi-automatic segmentation technique on every axial slice from the thoracic inlet to the beginning of the abdomen. First, an artificial intelligence-based algorithm segments the heart envelope. Then, a histogram-based method extracts two classes of pixels inside the envelope, corresponding to the EAT, expressed in cm^3^ (values between -190 and -30 Hounsfield units) and the soft tissue [7, 8, 13, 22, 24]. Supplementary Fig. 2 shows an example measurement of EAT volume. The intra- and inter-operator reproducibility of EAT volume measurements was 100% when tested on twenty patients.

### Statistical analyses

All statistical analyses were performed using R software (version 4.4.2). The distribution of each variable was assessed using the Shapiro–Wilk test to guide the choice of descriptive statistics and inferential methods. Quantitative variables are reported as mean ± standard deviation (SD), and categorical variables as counts and percentages.

Comparisons between participants with and without nephropathy were performed using Student's *t*-test or the Wilcoxon rank-sum test for continuous variables, as appropriate. For categorical variables, the χ^2^ test or Fisher's exact test was used when expected frequencies were small. For comparisons across more than two groups, one-way ANOVA or the Kruskal–Wallis test was applied, followed by Bonferroni-adjusted post-hoc tests when appropriate. Correlations between EAT volume and renal parameters (eGFR and ACR) were assessed using Pearson's or Spearman's rank correlation coefficient, depending on the normality of each variable.

To evaluate the independent association between EAT volume and renal outcomes, multivariable logistic regression models were fitted for three pre-specified outcomes: 1) presence of nephropathy (eGFR < 60 mL/min/1.73m^2^ and/or ACR ≥ 3 mg/mmol), 2) reduced eGFR (eGFR < 60 mL/min/1.73m^2^), and 3) albuminuria (ACR ≥ 3 mg/mmol). EAT volume was entered as a continuous predictor; results are presented as odds ratios (OR) per 10 cm^3^ increment, with 95% confidence intervals. Covariates were selected a priori based on clinical relevance and published literature, and included: age (per year), sex (women as reference), ethnicity (Caucasian as reference), diabetes duration (per year), HbA1c level (per %), hypertension, dyslipidaemia, BMI (per kg/m^2^), glucagon-like peptide 1 receptor agonist (GLP1-RA) use, sodium glucose cotransporter 2 inhibitor (SGLT2i) use, angiotensin-converting-enzyme (ACE) inhibitor or angiotensin II receptor blocker (ARB) use, and diuretic use. An additional exploratory model further including CRP was built to assess whether the observed associations with nephropathy might be driven by systemic inflammation.

Missing data for model covariates were handled using multiple imputation by chained equations (MICE package in R; m = 5 imputations, seed = 123). The imputation model included all variables entered in the regression models as well as the outcome variables. Pooled estimates were derived according to Rubin's rules. A complete-case analysis was performed as a supplementary analysis.

Given that the three outcomes were tested simultaneously on the same cohort and are inherently correlated, multiplicity correction was applied. Both Bonferroni correction (adjusted threshold: α = 0.05/3 ≈ 0.017) and Benjamini–Hochberg false discovery rate correction were applied as sensitivity analyses; the Benjamini–Hochberg procedure, which is more appropriate for correlated outcomes, is reported alongside raw p-values. Finally, given that participants were drawn from three distinct source studies, a sensitivity analysis was performed by including source study as a fixed effect in the multivariable model to assess potential heterogeneity. All tests were two-sided, and the primary significance threshold was set at *p* < 0.05.

## Results

### Patient characteristics

A total of 700 patients, of which 332 women, fulfilled inclusion criteria (Flow chart in Supplementary Fig. 1). Their characteristics are summarised in Table 1. They were 60.3 ± 10.4 (mean ± SD) years old and had type 2 diabetes for 14.6 ± 9.6 years. Nephropathy was observed in 295 patients (42.1%), including 87 (12.4%) with eGFR < 60 ml/min/1.73m^2^ and 258 (36.9%) with ACR ≥ 3 mg/mmol. As shown in Supplementary Table 1, the characteristics of the 700 individuals with type 2 diabetes who were included were similar to those of the 156 individuals who were not included. However, the GLP1-RA rate was higher among those included (35.6% vs. 24.5%, respectively; p < 0.01).

**Table 1:** Patient characteristics according to the presence of nephropathy

	Total N = 700	No nephropathy N = 405	Nephropathy N = 295	p	Available data
Age (years)	60.3 ± 10.4	58.9 ± 10.3	62.2 ± 10.3	< 0.001	700
Sex (Female)	332 (47.4%)	203 (50.1%)	129 (43.7%)	0.110	700
Body mass index (kg/m^2^)	30.2 ± 6.0	29.9 ± 5.8	30.5 ± 6.3	0.179	690
Body mass index status:			0.777 690		
< 25 kg/m^2^	123 (17.8%)	73 (18.2%)	50 (17.3%)		
25–29.9 kg/m^2^	258 (37.4%)	153 (38.2%)	105 (36.3%)		
≥ 30 kg/m^2^	309 (44.8%)	175 (43.6%)	134 (46.4%)		
Ethnic group:				0.080	698
North African	256 (36.7%)	150 (37.2%)	106 (35.9%)		
Caucasian	177 (25.4%)	95 (23.6%)	82 (27.8%)		
Sub-Sahara African/Antilles	154 (22.1%)	102 (25.3%)	52 (17.6%)		
Asiatic	94 (13.5%)	48 (11.9%)	46 (15.6%)		
Other	17 (2.4%)	8 (2.0%)	9 (3.1%)		
Diabetes duration (years)	14.6 ± 9.6	13.3 ± 9.2	16.4 ± 9.8	< 0.001	693
Hemoglobin A1c (%)	8.7 ± 2.1	8.5 ± 2.0	8.9 ± 2.2	0.034	641
*Diabetes-related treatment*					
Sulfonylurea or glinides	324 (46.4%)	189 (46.8%)	135 (45.9%)	0.881	698
Metformin	562 (80.4%)	333 (82.4%)	229 (77.6%)	0.138	699
Di-peptidyl-peptidase 4 inhibitor	202 (28.9%)	113 (28.0%)	89 (30.2%)	0.583	699
Alpha-glucosidase inhibitor	17 (2.4%)	10 (2.5%)	7 (2.4%)	1.000	699
Glucagon-like peptide 1 receptor agonist	171 (24.5%)	111 (27.5%)	60 (20.3%)	0.038	699
Sodium-glucose cotransporter-2 inhibitor	17 (2.4%)	6 (1.5%)	11 (3.7%)	0.098	699
Insulin	379 (54.1%)	217 (53.6%)	162 (54.9%)	0.785	700
Estimated glomerular filtration rate (mL/min/1.73m^2^)	91.7 ± 26.5	98.7 ± 20.6	82.1 ± 30.4	< 0.001	700
Estimated glomerular filtration rate classes				< 0.001	700
≥ 60 mL/min/1.73m^2^	613 (87.6%)	405 (100.0%)	208 (70.5%)		
< 60 mL/min/1.73m^2^	87 (12.4%)	0 (0.0%)	87 (29.5%)		
Albuminuria/creatininuria ratio (mg/mmol)	13.3 ± 43.8	0.9 ± 0.8	30.4 ± 63.7	< 0.001	700
Albuminuria/creatininuria ratio classes:				< 0.001	700
< 3 mg/mmol	442 (63.1%)	405 (100.0%)	37 (12.5%)		
3–29.9 mg/mmol	197 (28.1%)	0 (0.0%)	197 (66.8%)		
≥ 30 mg/mmol	61 (8.7%)	0 (0.0%)	61 (20.7%)		
Neuropathy	338 (49.0%)	187 (46.5%)	151 (52.4%)	0.146	690
Retinopathy	273 (39.7%)	141 (35.2%)	132 (46.0%)	0.005	688
Macroangiopathy	109 (15.6%)	49 (12.1%)	60 (20.5%)	0.004	697
Family history of premature coronary artery disease	53 (10.8%)	34 (11.9%)	19 (9.3%)	0.441	489
Hypertension	477 (68.4%)	235 (58.5%)	242 (82.0%)	< 0.001	697
Systolic blood pressure (mmHg)	135.2 ± 17.1	133.2 ± 16.3	137.8 ± 17.7	0.001	654
Diastolic blood pressure (mmHg)	76.9 ± 12.5	77.4 ± 12.5	76.2 ± 12.4	0.208	654
*Blood pressure lowering-treatment*					
ACE inhibitor or ARB	430 (61.6%)	207 (51.4%)	223 (75.6%)	< 0.001	698
Beta blocker	115 (16.5%)	44 (10.9%)	71 (24.1%)	< 0.001	698
Calcium channel inhibitor	203 (29.1%)	90 (22.3%)	113 (38.3%)	< 0.001	698
Furosemide	24 (4.5%)	6 (1.9%)	18 (8.4%)	0.001	531
Spironolactone	15 (2.8%)	5 (1.6%)	10 (4.7%)	0.067	531
Other diuretics	271 (38.8%)	129 (32.0%)	142 (48.1%)	< 0.001	698
Other antihypertensives	108 (15.5%)	47 (11.7%)	61 (20.7%)	0.002	698
Dyslipidemia	465 (66.7%)	247 (61.4%)	218 (73.9%)	0.001	697
Total Cholesterol (mmol/L)	4.2 ± 1.1	4.2 ± 1.1	4.2 ± 1.2	0.608	690
HDL cholesterol (mmol/L)	1.2 ± 0.3	1.2 ± 0.4	1.1 ± 0.3	0.001	693
Triglycerides (mmol/L)	1.7 ± 1.1	1.6 ± 1.0	1.9 ± 1.2	< 0.001	693
LDL cholesterol (mmol/L)	2.3 ± 0.9	2.3 ± 0.9	2.2 ± 1.0	0.600	685
*Lipid lowering-treatment*					
Statin	437 (62.6%)	239 (59.3%)	198 (67.1%)	0.043	698
Fibrate	23 (3.3%)	11 (2.7%)	12 (4.1%)	0.445	698
Ezetimibe	31 (4.4%)	16 (4.0%)	15 (5.1%)	0.603	698
Current smoking	103 (14.8%)	60 (14.9%)	43 (14.7%)	1.000	694
C-reactive protein (mg/L)	6.5 ± 19.4	5.5 ± 15.0	7.9 ± 24.3	0.139	675
Epicardial adipose tissue volume (cm^3^)	98.0 ± 38.4	93.8 ± 36.8	103.6 ± 39.8	0.001	700
Coronary artery calcium score (AU)	193.4 ± 438.6	128.8 ± 295.5	282.5 ± 569.3	< 0.001	699
Coronary artery calcium score > 100 AU	239 (34.3%)	103 (25.6%)	136 (46.3%)	< 0.001	696

Data are presented as the mean ± standard deviation or n (%). ACE: Angiotensin-converting-enzyme. ARB: angiotensin II receptor blocker, AU: Agatston unit.

p-value: comparison between patients with and without nephropathy

### Parameters associated with nephropathy

Compared to those without nephropathy, patients with nephropathy (Table 1) were older, had a longer diabetes duration and higher HbA1c levels and were more likely to have diabetes-related complications like retinopathy, macroangiopathy and higher CAC score. They were more likely to have hypertension and blood pressure lowering-treatment, with higher systolic blood pressure; and to have dyslipidemia and statin treatment, with lower HDL-cholesterol and higher triglyceride levels.

### Parameters associated with glomerular filtration rate

Supplementary Table 2 shows the characteristics of the patients by eGFR < or ≥ 60 ml/min/1.73m^2^. eGFR < 60 ml/min/1.73m^2^ was associated with higher age, higher BMI, longer diabetes duration, lower HbA1c level, higher CAC score and more hypertension, dyslipidaemia and smoking. They also were more likely to have macroangiopathy whereas others diabetes related complications were similar between groups.

### Parameters associated with albuminuria

Supplementary Table 3 shows the characteristics of the patients by ACR classes. Higher ACR was more frequent with increasing age, diabetes duration and HbA1c levels, in men than in women and in individuals with diabetes-related complications (retinopathy, macroangiopathy, high CAC score), hypertension or dyslipidaemia. Those patients were more likely to be treated by insulin and blood pressure lowering treatment as well as diuretic, and less by metformin and sulfonylurea.

### Epicardial adipose tissue volume and nephropathy

**Fig. 1:**
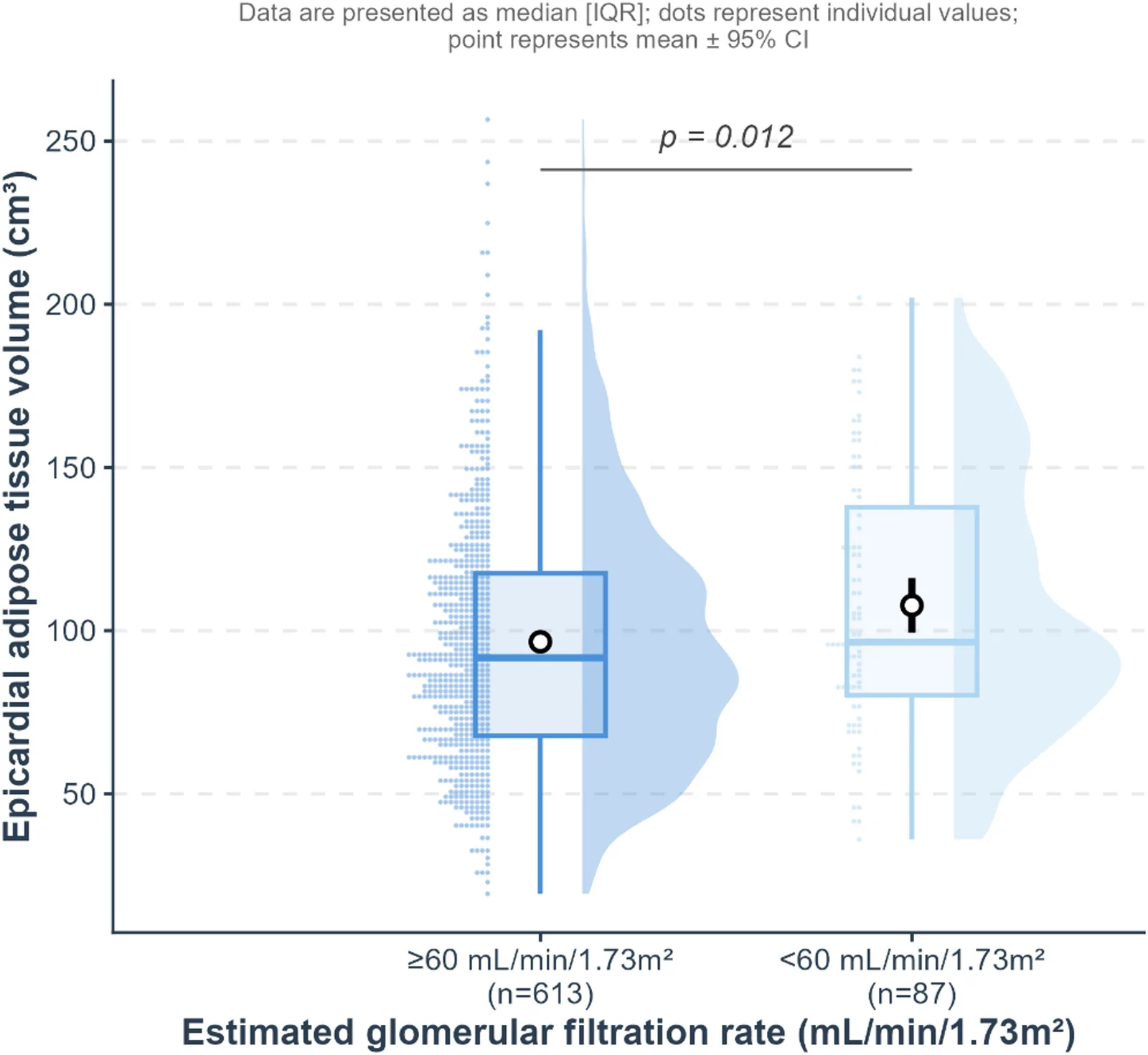
Epicardial adipose tissue volume by estimated glomerular filtration rate classes

**Fig. 2:**
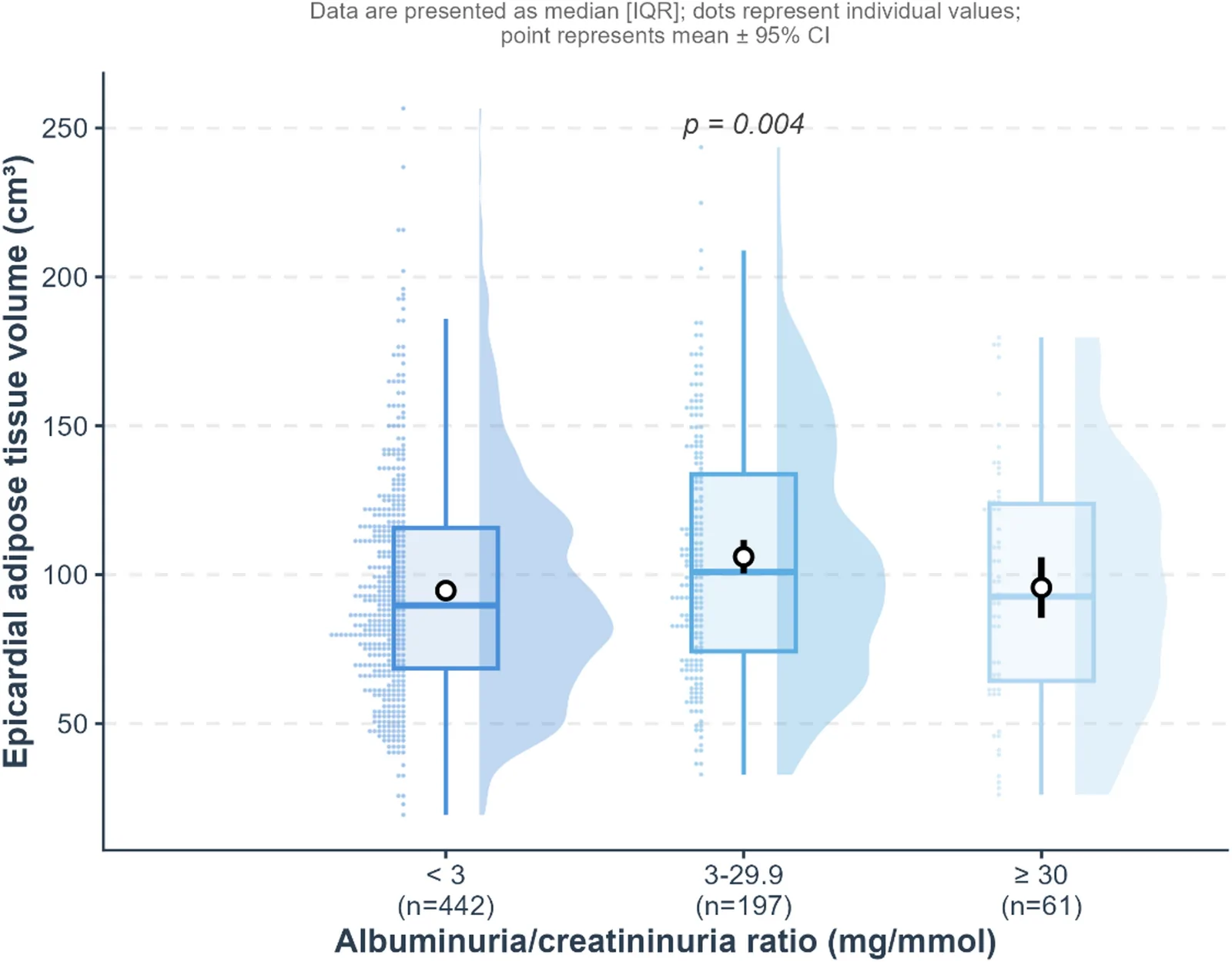
Epicardial adipose tissue volume by albuminuria/creatininuria ratio classes

EAT volume was higher in patients with nephropathy compared to those without (respectively: 103.6 versus 93.8 cm^3^, p = 0.001) (Table 1). Figures 1 and 2 showed that EAT volume differed by eGFR and ACR classes, respectively (mean ± SD in supplementary tables 2 and 3).

**Fig. 3:**
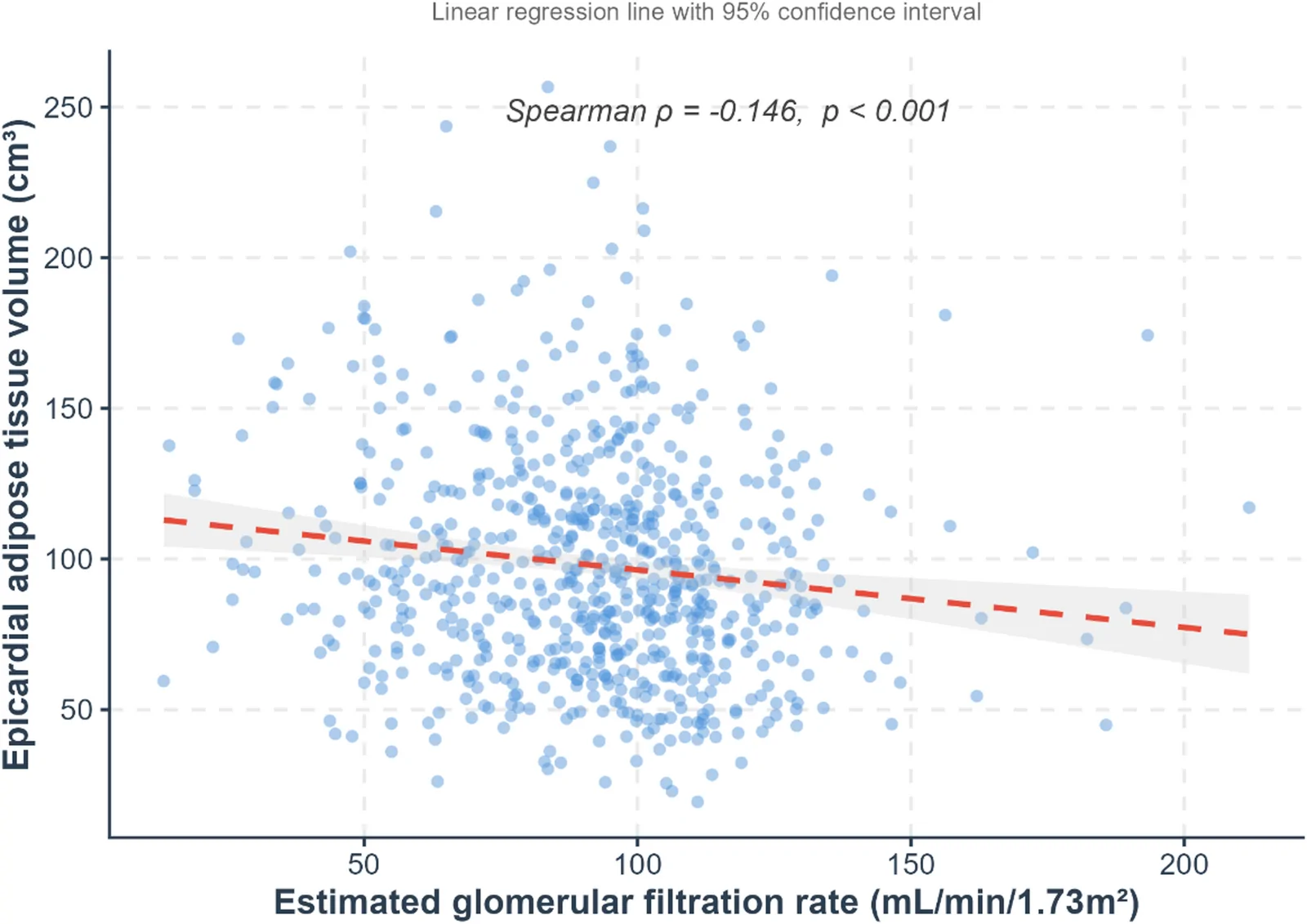
Correlation between epicardial adipose tissue volume and estimated glomerular filtration rate

**Fig. 4:**
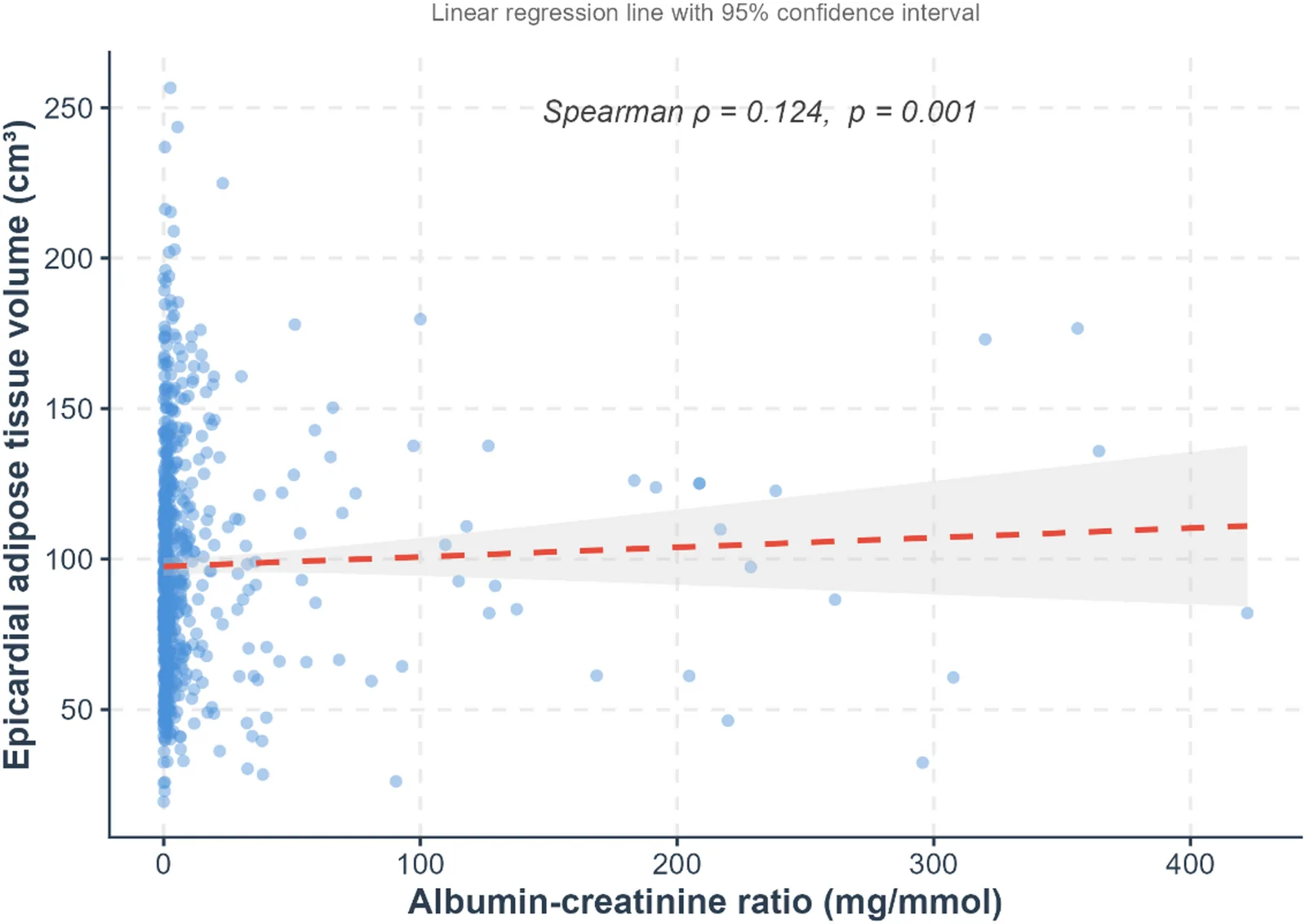
Correlation between epicardial adipose tissue volume and albuminuria / creatininuria ratio

Moreover, EAT volume was weakly and negatively correlated to eGFR (r = − 0.146; p < 0.001) (Fig. 3) and weakly and positively correlated to ACR (r = 0.124; p = 0.001) (Fig. 4).

To note, EAT volume was weakly but positively correlated with C reactive protein (r = 0.089; p = 0.020; Supplementary Fig. 3).

Multivariable analysis (Table 2) showed that each 10 cm^3^ increase in EAT volume was independently associated with a 5.8% increase in the odds of nephropathy (OR = 1.058 [95% CI 1.007–1.112], p = 0.026) and a 5.9% increase in the odds of albuminuria (ACR ≥ 3 mg/mmol; OR = 1.059 [95% CI 1.008–1.114], p = 0.024), although odds ratios were close to unity and confidence intervals were narrow. However, EAT volume was not independently associated with reduced eGFR (eGFR < 60 mL/min/1.73m^2^; OR = 1.047 [95% CI 0.975–1.123], p = 0.204). After Benjamini–Hochberg correction, the associations between EAT volume and nephropathy (BH-adjusted p = 0.040) and albuminuria (BH-adjusted p = 0.040) remained statistically significant. The addition of CRP to the model did not materially alter these findings (nephropathy: OR = 1.058 [95% CI 1.007–1.112], p = 0.027; Supplementary Table 4). A sensitivity analysis including source study [7, 8, 13] as a fixed effect yielded consistent results: the association with nephropathy (OR = 1.058 [95% CI 1.007–1.112], p = 0.027) and albuminuria (OR = 1.062 [95% CI 1.007–1.113], p = 0.026) remained significant, while the association with eGFR < 60 mL/min/1.73m^2^ showed a similar point estimate (OR = 1.053) but did not reach statistical significance (p = 0.153), consistent with our primary findings (Supplementary Table 5). Finally, the results of the complete-case analysis were consistent with the main multiple imputation analysis (Supplementary Table 6).

**Table 2:** Association between epicardial adipose tissue volume and nephropathy and components in multivariable analyses

Adjustment for	Outcomes	Odds ratio (by 10 cm^3^ of EAT)	[95% CI]	p-value
Age (per year) Gender (Ref: Female) Ethnicity (Ref: Caucasian) Diabetes duration (per year) HbA1c level (per %) Hypertension Dyslipidaemia Body mass index (kg/m^2^) GLP1-RA SGLT2i Diuretic use ACE inhibitor or ARB	Nephropathy	1.058	[1.007–1.112]	**0.026**
	eGFR < 60 mL/min/1.73m^2^	1.047	[0.975–1.123]	0.204
	ACR ≥ 3 mg/mmol	1.059	[1.008–1.114]	**0.024**

ACR: albuminuria/creatininuria ratio, ACE: Angiotensin-converting enzyme, ARB: angiotensin II receptor blocker, CI: confidence interval, EAT: epicardial adipose tissue; eGFR: estimated glomerular filtration rate, GLP1-RA: Glucagon-like peptide-1 receptor agonist, SGLT2i: Sodium/Glucose cotransporter 2 inhibitor. Bold depicts statistical significance

## Discussion

### Main result

Our study showed that, independently of principal confounding factors, there was a weak positive association between EAT volume and nephropathy in people living with type 2 diabetes. In detail, the association was only significant for ACR.

### Comparison with literature dealing with eGFR and ACR

We did not find any studies that specifically explored the association between EAT amount and eGFR. Some [8, 9, 12, 25, 26] but not all studies [27] also showed a negative correlation between those two parameters. The latter study [27] included a particular population, i.e. only patients with eGFR < 45 mL/min/1.73m^2^. Therefore, our study is the first to categorise patients according to their eGFR, a fortiori, in a multivariable analysis. We have found that the negative association between EAT volume and eGFR in univariate analysis was no longer present after adjustment for confounders. We hypothesize that it is because of the presence in the multivariable model of age, presence of hypertension and BMI, which are known to be involved in the decrease of eGFR [28].

Considering ACR now, the present study showed a positive correlation and significant association between EAT volume and ACR, even after adjusting for major confounding factors. Upon closer inspection, we observed a non-linear relationship between EAT and ACR in the categorical association analyses. EAT volume was not highest among individuals with ACR ≥ 30 mg/mmol. These patients had a higher use rate of anti-hypertensive treatments, including ACE inhibitors, ARB and diuretics. These three variables were considered in the multivariable analyses and are not known to reduce EAT volume. Conversely, we found that they were less likely to be treated with GLP1-RA and were treated similarly with SGLT2i. While these treatments have been shown to reduce EAT volume [29–31]. they cannot explain why EAT volume was not the highest in individuals with ACR ≥ 30 mg/mmol. In the literature, three studies, although not originally dedicated to explore the association between EAT amount and diabetic nephropathy, had already suggested an association between an increase of EAT amount in patients with diabetes and increased ACR [8, 9, 27]. Two other studies specifically explored, like ours, the association between EAT amount and ACR status [20, 21]. In the first one, including 200 patients living with type 2 diabetes [20], the association between EAT amount and ACR was stronger than ours (r = 0.318; p < 0.001) and persisted after adjustment (p = 0.017). However, (i) it evaluated EAT thickness by echocardiography rather than EAT volume, which is known to give a better volumetric EAT estimate [32] and (ii) variables used for adjustment did not include hypertension, whereas this condition is known to be a major risk factor of increased ACR [33]. The second study had similar results compared to ours: in a population of 180 patients with type 2 diabetes, EAT thickness was higher in patients with albuminuria compared to those without (respectively: EAT = 6.5 ± 1.4 (mean ± SD); 5.9 ± 1.4; p = 0.014), but this association disappeared after adjustment for confounders [21].

### Real mechanism or surrogate marker of visceral adiposity?

The pathogenesis of diabetic nephropathy is multifactorial and involves haemodynamic abnormalities and metabolic disorders such as advanced glycation end (AGE) products synthesis and oxidative stress [34, 35]. Those disorders both promote the synthesis of pro-inflammatory cytokines and growth factors by inflammatory cells, i.e. monocytes and macrophages [34]. Among pro-inflammatory proteins, tumor necrosis factor (TNF), transforming growth factor β (TGF-β) and interleukin 6 (IL-6) have been found to be increased in patients living with type 2 diabetes and to play a role in the development and the progression of diabetic nephropathy [35]. This disease is thus closely related to inflammation, leading to expansion of the mesangial matrix and glomerulosclerosis and then to the apparition of albuminuria and decreasing of the GFR [34].

On the other hand, EAT is part of visceral adipose tissue with properties on thermogenesis, mechanical protection for coronary arteries, and metabolism by providing free fatty acids to the myocardium [14, 36, 37]. Its role in cardiovascular risk and outcomes has already been well described [7, 14, 36, 38–40]. This tissue contains many resident immune cells, described as more pro-inflammatory than usual, i.e. more M1 than M2 and fewer regulatory T-cells (14, 36). EAT is also known to synthesise mediators, mostly involved in the regulation of endothelial function, coagulation and inflammation [38] but also adipokines such as leptin and adiponectin [6, 36]. These secretions can either protect or alter the health of the myocardium and coronary arteries [36]. In pathological conditions, the secretion of pro-inflammatory or proatherogenic factors increases [6, 37]. Secreted mediators act locally in two ways: paracrine and vasocrine [6, 36, 39].

However, a systemic secretion by EAT has been controversial and does not seem to exist [41]. Thus, this would mean that the association we found between EAT volume and nephropathy cannot be explained by a systemic secretion. Then, how can EAT be linked to a non-cardiac condition such as nephropathy? EAT would only be a surrogate marker of global visceral adiposity in this population. General obesity has already been considered as a predictor of chronic kidney disease [28], but only few studies assess specific fat depots. An increase in EAT volume might coincide with an increase in other fat depots [42, 43]: near the heart such as pericardial adipose tissue (PAT) [44, 45] or near the kidney such as perirenal adipose tissue (PRAT) [46, 47]. First, PAT is located outside the parietal pericardium, without direct contact with the myocardium [45]. Like EAT, PAT has been associated with various cardiac diseases [44, 45]. Unlike EAT, it has also shown a role in systemic inflammation, insulin resistance, and endothelial dysfunction; pathways also found in nephropathy development and progression [44]. Despite these potential links, to date, no studies have shown a link between PAT and nephropathy in people with diabetes. On the other hand, PRAT is a visceral adipose tissue located in the retroperitoneal space surrounding the kidney and is metabolically active [47, 48], as is EAT. Several studies have already shown a relation between PRAT increase and kidney disease [46, 48–50], including in patients living with type 2 diabetes [42, 51, 52]. More importantly, involved mechanisms seem to be similar to those from EAT, by locally secreting adipokines and pro-inflammatory proteins such as TNF-a and IL-6, promoting oxidative stress and renal microvascular damage [39, 46–48], which impact the development of nephropathy. Thus, inflammation might be closely related with EAT, PAT and PRAT as well as nephropathy as we previously discussed. However, in the present study, we demonstrated that the association between EAT volume and nephropathy remained significant when a single CRP measurement was incorporated into the model. Therefore, further specific studies are needed.

### Strengths and limitations

Our study is the first to stratify patients depending on their renal status using both eGFR and ACR. However, only a single measurement of ACR and eGFR was available. Also, thanks to a large cohort, we were able to adjust our analysis for possible confounders, which empowers our results. However, residual confounding possible as other cause of nephropathy or adiposity distribution were unknown. We measured EAT volume rather than thickness, with a perfect intra- and inter-operator reproducibility. However, CT examinations were performed using scanners from different manufacturers (GE and Siemens).

Our study had several additional limitations. First, the monocentric design of the study, the specific hospital setting and the fact that patients were mainly included in primary prevention (where cardiovascular evaluation included the measurement of CAC score) might influence the generalisability of the findings. Second, due to the insufficient number of patients with other types of diabetes, we could not study these populations. Because of its cross-sectional design, we cannot infer cause-and-effect relationships from our findings. Again, no mechanistic biomarkers beyond CRP were available and no other visceral tissue except EAT was measured. The number of events per variable was verified for all outcomes and was satisfactory for nephropathy (EPV = 22.7) and albuminuria (EPV = 19.8), but borderline for eGFR < 60 mL/min/1.73m^2^ (EPV = 6.8, below the recommended threshold of 10), which should be considered when interpreting results for this specific outcome.

### Perspectives

Our results need to be validated by complementary studies. As an example, a study that evaluates the PRAT volume and its link with diabetic nephropathy independently of the EAT would be interesting. If the results are confirmed, this would stir up hypotheses. First, what is the respective part of each fat deposit (PAT or PRAT) in the development of nephropathy and secondly which one comes first between fat deposition and nephropathy.

Studies have shown that GLP1-RA and SGLT2i also reduce the volume of EAT [29–31] and more recent studies suggest the same for PRAT volume [52, 53]. It would be interesting to know if their nephroprotective effect is mediated by a reduction in inflammation linked to the reduction of the volume of the PAT/PRAT.

## Conclusion

Using a large cohort and after adjusting for confounders, we found an association between EAT volume and nephropathy, especially ACR, in patients living with type 2 diabetes. This association may be partly explained by systemic inflammation and metabolic mechanisms, potentially driven by EAT and/or other associated ectopic fat depots in persons with type 2 diabetes. Drawing on the literature and our study, EAT seems to be a surrogate marker of global visceral adiposity – with others adipose depots like PAT or PRAT – which has already been linked to chronic kidney disease. Further investigation, ideally with adipose depots distribution and other inflammation biomarkers data, is needed to fully understand the underlying mechanisms.

## Supplementary Information

Supplementary Material 1.

## Data Availability

Data for the present analysis can be provided from the corresponding author on reasonable request.

## Abbreviations

ACE: Angiotensin-converting-enzyme
ACR: Albuminuria/creatininuria ratio
AGE: Advanced glycation end [products]
ARB: Angiotensin II receptor blocker
AU: Agatston unit
BMI: Body mass index
CAC: Coronary artery calcium
CRP: C-reactive protein
CT: Computed tomography
CI: Confidence interval
EAT: Epicardial adipose tissue
eGFR: Estimated glomerular filtration rate
GLP1-RA: Glucagon-like peptide 1 receptor agonist
HbA1c: Glycated hemoglobin
OR: Odds ratio
PAT: Pericardial adipose tissue
PRAT: Perirenal adipose tissue
SD: Standard deviation
SGLT2i: Sodium glucose transporter 2 inhibitor
TNF: Tumor necrosis factor
TGF-β: Transforming growth factor β
IL-6: Interleukin 6
