# Supplementary material for: Epicardial adipose tissue volume and nephropathy in people living with type 2 diabetes: the Kidn-EAT study
Source: Cardiovasc Diabetol. 2026 Jul 30;25:312. doi: 10.1186/s12933-026-03311-z (PMC13711880; doi:10.1186/s12933-026-03311-z)
Supplement: Supplementary file 1 [file 12933_2026_3311_MOESM1_ESM.docx]

## Supplementary data:

Supplementary Figure 1: Flowchart of the study

Supplementary Figure 2: Characterization of epicardial adipose tissue volume

a: three-dimension reconstitution of epicardial adipose tissue; example of axial image before (b) and after (c) epicardial adipose tissue identification; example of coronal image before (d) and after (e) epicardial adipose tissue identification

Supplementary Figure 3: Correlation between EAT volume and C-reactive protein level

Supplementary Table 1: Comparison of patients with type 2 diabetes who were included and excluded in the study

|  | **Excluded (N=156)** | **Included (N=700)** | **p** | **N** |
| --- | --- | --- | --- | --- |
| Age (years) | 61.0 [55.0;66.5] | 61.0 [54.0;68.0] | 0.886 | 856 |
| Sex (Female): | 73 (45.6%) | 332 (47.4%) | 0.746 | 856 |
| Body mass index (kg/m²) | 28.9 [26.0;33.0] | 29.0 [26.0;33.6] | 0.486 | 846 |
| Ethnicity: |  |  | 0.105 | 854 |
| Asiatic | 22 (13.8%) | 94 (13.5%) |  |  |
| Caucasian | 50 (31.4%) | 177 (25.4%) |  |  |
| North African | 49 (30.8%) | 256 (36.7%) |  |  |
| Other | 0 (0.0%) | 17 (2.4%) |  |  |
| Sub-Sahara African/Antilles | 38 (23.9%) | 154 (22.1%) |  |  |
| Diabetes duration (years) | 13.0 [7.0;18.5] | 14.0 [8.0;20.0] | 0.289 | 850 |
| HbA1c (%) | 7.8 [7.0;9.6] | 8.3 [7.1;9.8] | 0.116 | 773 |
| Epicardial adipose tissue volume (cm³) | 88.4 [68.7;116.7] | 92.6 [69.4;120.8] | 0.201 | 856 |
| Hypertension: | 108 (67.5%) | 477 (68.4%) | 0.892 | 856 |
| Dyslipidemia: | 112 (70.4%) | 465 (66.7%) | 0.418 | 856 |
| Diabetes related treatments: |  |  |  |  |
| Sulfonylurea or glinides: | 73 (45.6%) | 324 (46.4%) | 0.925 | 854 |
| Metformin: | 124 (77.5%) | 562 (80.4%) | 0.474 | 855 |
| Glucagon-like peptide 1 receptor agonist: | 57 (35.6%) | 171 (24.5%) | 0.005 | 855 |
| Sodium-glucose cotransporter-2 inhibitor: | 7 (4.4%) | 17 (2.4%) | 0.185 | 855 |
| Estimated glomerular filtration rate (mL/min/1.73m²) | 90.9 [70.1;105.6] | 94.0 [76.0;107.8] | 0.201 | 848 |
| Albumin-creatinine ratio (mg/mmol) | 4.2 [1.4;20.2] | 1.6 [0.5;6.3] | 0.157 | 707 |
| Neuropathy: | 85 (53.8%) | 338 (49.0%) | 0.316 | 846 |
| Retinopathy: | 58 (36.9%) | 273 (39.7%) | 0.587 | 844 |
| Macroangiopathy: | 24 (15.0%) | 109 (15.6%) | 0.936 | 853 |
| ACE inhibitor or ARB: | 101 (63.1%) | 430 (61.6%) | 0.790 | 854 |
| Current smoking: | 17 (14.9%) | 73 (13.7%) | 0.860 | 645 |
| C-reactive protein (mg/L) | 3.0 [1.0;5.0] | 3.0 [1.0;5.0] | 0.947 | 826 |
| Coronary artery calcium score (AU) | 10.9 [0.0;150.5] | 21.0 [0.0;196.5] | 0.444 | 855 |
| Coronary artery calcium score > 100 AU: | 49 (31.0%) | 239 (34.3%) | 0.481 | 852 |

*Data are presented as the median +- [IQR25; IQR75] or n (%). ACE: Angiotensin-converting-enzyme. ARB: angiotensin II receptor blocker. AU: Agatston unit.*

Supplementary Table 2: Patient characteristics according to glomerular filtration rate classes

|  | Total  N = 700 | *≥* 60 mL/min/1.73m²  N = 613 | < 60 mL/min/1.73m²  N = 87 | p | Available data | | |
| --- | --- | --- | --- | --- | --- | --- | --- |
| Age (years) | 60.3 ±10.4 | 59.3 ±10.1 | 67.0 ±9.7 | <0.001 | 700 | | |
| Sex (F) | 332 (47.4%) | 283 (46.2%) | 49 (56.3%) | 0.097 | 700 | | |
| Body mass index (kg/m²) | 30.2 ±6.0 | 29.8 ±5.8 | 32.4 ±7.2 | 0.002 | 690 | | |
| Body mass index status: |  |  |  | 0.056 | 690 | | |
| *<* 25 kg/m² | 123 (17.8%) | 114 (18.9%) | 9 (10.3%) |  |  | | |
| 25–29.9 kg/m² | 258 (37.4%) | 228 (37.8%) | 30 (34.5%) |  |  | | |
| *≥* 30 kg/m² | 309 (44.8%) | 261 (43.3%) | 48 (55.2%) |  |  | | |
| Origin: |  |  |  | 0.022 | 698 | | |
| North African | 256 (36.7%) | 223 (36.5%) | 33 (37.9%) |  |  | | |
| Caucasian | 177 (25.4%) | 151 (24.7%) | 26 (29.9%) |  |  | | |
| Sub-Sahara African/Antilles | 154 (22.1%) | 130 (21.3%) | 24 (27.6%) |  |  | | |
| Asiatic | 94 (13.5%) | 90 (14.7%) | 4 (4.6%) |  |  | | |
| Other | 17 (2.4%) | 17 (2.8%) | 0 (0.0%) |  |  | | |
| Diabetes duration (years) | 14.6 ±9.6 | 14.0 ±9.3 | 19.3 ±10.7 | <0.001 | 693 | | |
| Hemoglobin A1c (%) | 8.7 ±2.1 | 8.7 ±2.1 | 8.2 ±2.0 | 0.028 | 641 | | |
| Diabetes-related treatments: |  |  |  |  |  | | |
| Sulfonylurea or glinides | 324 (46.4%) | 295 (48.3%) | 29 (33.3%) | 0.012 | 698 | | |
| Metformin | 562 (80.4%) | 505 (82.5%) | 57 (65.5%) | *<*0.001 | 699 | | |
| Di-peptidyl-peptidase 4 inhibitor | 202 (28.9%) | 181 (29.6%) | 21 (24.1%) | 0.357 | 699 | | |
| Alpha-glucosidase inhibitor | 17 (2.4%) | 16 (2.6%) | 1 (1.3%) | 0.710 | 699 | | |
| Glucagon-like peptide 1 receptor agonists | 171 (24.5%) | 158 (25.8%) | 13 (14.9%) | 0.038 | 699 | | |
| Sodium-glucose cotransporter-2 inhibitor | 17 (2.4%) | 12 (2.0%) | 5 (5.7%) | 0.049 | 699 | | |
| Insulin | 379 (54.1%) | 327 (53.3%) | 52 (59.8%) | 0.312 | 700 | | |
| Estimated glomerular filtration rate (mL/min/1.73m²) | 91.7 ±26.5 | 98.2 ±21.1 | 46.1 ±11.3 | <0.001 | 700 | | |
| Albuminuria / creatininuria ratio (mg/mmol) | 13.3 ±43.8 | 8.5 ±29.8 | 47.1 ±89.4 | <0.001 | 700 | | |
| Retinopathy | 273 (39.7%) | 233 (38.6%) | 40 (47.1%) | 0.172 | 688 | | |
| Neuropathy | 338 (49.0%) | 290 (48.1%) | 48 (55.2%) | 0.263 | 690 | | |
| Macroangiopathy | 109 (15.6%) | 88 (14.4%) | 21 (24.1%) | 0.030 | 697 | | |
| Family history of premature coronary artery disease | 53 (10.8%) | 48 (11.2%) | 5 (8.3%) | 0.657 | 489 | | |
| Hypertension | 477 (68.4%) | 392 (64.3%) | 85 (97.7%) | *<*0.001 | 697 | | |
| Systolic blood pressure (mmHg) | 135.2 ±17.1 | 134.7 ±16.7 | 138.9 ±19.3 | 0.064 | 654 | | |
| Diastolic blood pressure (mmHg) | 76.9 ±12.5 | 77.4 ±12.4 | 73.2 ±12.3 | 0.005 | 654 | | |
| Hypertension-related treatments: |  |  |  |  |  | | |
| ACE inhibitor or ARB | 430 (61.6%) | 353 (57.8%) | 77 (88.5%) | *<*0.001 | 698 | | |
| Beta blocker | 115 (16.5%) | 79 (12.9%) | 36 (41.4%) | *<*0.001 | 698 | | |
| Calcium channel inhibitor | 203 (29.1%) | 156 (25.5%) | 47 (54.0%) | *<*0.001 | 698 | | |
| Furosemide | 24 (4.5%) | 13 (2.8%) | 11 (18.3%) | *<*0.001 | 531 | | |
| Spironolactone | 15 (2.8%) | 8 (1.7%) | 7 (11.7%) | 0.001 | 531 | | |
| Other diuretics | 271 (38.8%) | 215 (35.2%) | 56 (64.4%) | *<*0.001 | 698 | | |
| Other antihypertensives | 108 (15.5%) | 74 (12.1%) | 34 (39.1%) | *<*0.001 | 698 | | |
| Dyslipidemia | 465 (66.7%) | 394 (64.6%) | 71 (81.6%) | 0.002 | 697 | | |
| Total cholesterol (mmol/L) | 4.2 ±1.1 | 4.2 ±1.2 | 4.2 ±1.1 | 0.789 | 690 | | |
| HDL cholesterol (mmol/L) | 1.2 ±0.3 | 1.2 ±0.3 | 1.2 ±0.4 | 0.414 | 693 | | |
| Triglycerides (mmol/L) | 1.7 ±1.1 | 1.7 ±1.1 | 1.8 ±1.0 | 0.465 | 693 | | |
| LDL cholesterol (mmol/L) | 2.3 ±0.9 | 2.3 ±1.0 | 2.1 ±0.9 | 0.129 | 685 | | |
| Dyslipidemia-related treatments: |  |  |  |  |  | | |
| Statin | 437 (62.6%) | 371 (60.7%) | 66 (75.9%) | 0.009 | 698 | | |
| Fibrate | 23 (3.3%) | 20 (3.3%) | 3 (3.4%) | 1.000 | 698 | | |
| Ezetimibe | 31 (4.4%) | 23 (3.8%) | 1 (9.2%) | 0.044 | 698 | | |
| Current smoking | 103 (14.8%) | 100 (16.4%) | 2 (2.7%) | 0.002 | 694 | | |
| C-reactive protein (mg/L) | 6.5 ±19.4 | 6.7 ±20.6 | 5.1 ±7.1 | 0.173 | 675 | | |
| Epicardial adipose tissue (cm^3^) | 98.0 ±38.4 | 96.6 ±38.1 | 107.7 ±39.2 | 0.012 | 700 | | |
| Coronary artery calcium score (AU) | 193.4 ±438.6 | 173.4 ±394.4 | 334.5 ±658.0 | 0.028 | 699 | | |
| Coronary artery calcium score > 100 AU | 239 (34.3%) | 195 (32.0%) | 44 (50.6%) | 0.001 | 696 | | |
|  |  |  |  |  | |  |  |

*Data are presented as the mean ± standard deviation or n (%). ACE: Angiotensin-converting-enzyme, ARB: angiotensin II receptor blocker, AU: Agatston unit*

*p-value: comparison between the three classes*

Supplementary Table 3: Patient characteristics according to albuminuria/creatininuria ratio classes

|  | Total  N=700 | <3 mg/mmol  N=442 | 3 – 29.9 mg/mmol  N=197 | ≥ 30 mg/mmol  N=61 | p | Available data |
| --- | --- | --- | --- | --- | --- | --- |
| Age (years) | 60.3 ±10.4 | 59.5 ±10.5 | 61.6 ±9.8 | 61.6 ±11.5 | 0.043 | 700 |
| Sex (F) | 332 (47.4%) | 228 (51.6%) | 79 (40.1%) | 25 (41.0%) | 0.016 | 700 |
| Body mass index (kg/m²) | 30.2 ±6.0 | 30.3±6.2 | 29.9 ±5.7 | 30.4 ±6.1 | 0.686 | 690 |
| Body mass index status: |  |  |  |  | 0.746 | 690 |
| *<* 25 kg/m² | 123 (17.8%) | 76 (17.4%) | 36 (18.7%) | 11 (18.6%) | | |
| 25–29.9 kg/m² | 258 (37.4%) | 164 (37.4%) | 76 (39.4%) | 18 (30.5%) | | |
| *≥* 30 kg/m² | 309 (44.8%) | 198 (45.2%) | 81 (42.0%) | 30 (50.8%) |  |  |
| Origin: |  |  |  |  | . | 698 |
| North African | 256 (36.7%) | 162 (36.8%) | 68 (34.5%) | 26 (42.6%) | | |
| Caucasian | 177 (25.4%) | 107 (24.3%) | 57 (28.9%) | 13 (21.3%) | | |
| Sub-Sahara African/Antilles | 154 (22.1%) | 114 (25.9%) | 29 (14.7%) | 11 (18.0%) | | |
| Asiatic | 94 (13.5%) | 49 (11.1%) | 34 (17.3%) | 11 (18.0%) | | |
| Other | 17 (2.4%) | 8 (1.8%) | 9 (4.6%) | 0 (0.0%) | | |
| Diabetes duration (years) | 14.6 ±9.6 | 13.7 ±9.3 | 15.4 ±9.2 | 19.0 ±11.3 | <0.001 | 693 |
| Hemoglobin A1c (%) | 8.7 ±2.1 | 8.5 ±2.0 | 9.0 ±2.2 | 8.8 ±2.2 | 0.012 | 641 |
| Diabetes-related treatment: |  |  |  |  |  |  |
| Sulfonylurea or glinides | 324 (46.4%) | 206 (46.7%) | 100 (51.0%) | 18 (29.5%) | 0.013 | 698 |
| Metformin | 562 (80.4%) | 362 (82.1%) | 160 (81.2%) | 40 (65.6%) | 0.009 | 699 |
| Di-peptidyl-peptidase 4 inhibitor | 202 (28.9%) | 125 (28.3%) | 67 (34.0%) | 10 (16.4%) | 0.027 | 699 |
| Alpha-glucosidase inhibitor | 17 (2.4%) | 11 (2.5%) | 5 (2.5%) | 1 (1.6%) | 1.000 | 699 |
| Glucagon-like peptide 1 receptor agonists | 171 (24.5%) | 119 (27.0%) | 45 (22.8%) | 7 (11.5%) | 0.025 | 699 |
| Sodium-glucose cotransporter-2 inhibitor | 17 (2.4%) | 8 (1.8%) | 6 (3.0%) | 3 (4.9%) | 0.179 | 699 |
| Insulin | 379 (54.1%) | 234 (52.9%) | 102 (51.8%) | 43 (70.5%) | 0.026 | 700 |
| Estimated glomerular filtration rate (mL/min/1.73m²) | 91.7 ±26.5 | 94.4 ±24.4 | 90.9 ±27.1 | 74.8 ±32.3 | <0.001 | 700 |
| Albuminuria / creatininuria ratio (mg/mmol) | 13.3 ±43.8 | 0.9 ±0.8 | 8.9 ±6.0 | 117.5 ±100.1 | <0.001 | 700 |
| Retinopathy | 273 (39.7%) | 154 (35.3%) | 81 (42.4%) | 38 (62.3%) | *<*0.001 | 688 |
| Neuropathy | 338 (49.0%) | 204 (46.5%) | 104 (54.7%) | 30 (49.2%) | 0.163 | 690 |
| Macroangiopathy | 109 (15.6%) | 57 (12.9%) | 41 (21.0%) | 11 (18.0%) | 0.030 | 697 |
| Family history of premature coronary artery disease | 53 (10.8%) | 36 (11.5%) | 14 (10.2%) | 3 (7.5%) | 0.803 | 489 |
| Hypertension | 477 (68.4%) | 270 (61.5%) | 155 (78.7%) | 52 (85.2%) | *<*0.001 | 697 |
| Systolic blood pressure (mmHg) | 135.2 ±17.1 | 133.4 ±16.9 | 137.6 ±17.5 | 140.0 ±15.5 | 0.002 | 654 |
| Diastolic blood pressure (mmHg) | 76.9 ±12.5 | 77.0 ±12.6 | 77.2 ±12.4 | 74.9 ±11.7 | 0.453 | 654 |
| Hypertension-related treatment: |  |  |  |  |  |  |
| ACE inhibitor or ARB | 430 (61.6%) | 236 (53.6%) | 144 (73.1%) | 50 (82.0%) | *<*0.001 | 698 |
| Beta blocker | 115 (16.5%) | 56 (12.7%) | 37 (18.8%) | 22 (36.1%) | *<*0.001 | 698 |
| Calcium channel inhibitor | 203 (29.1%) | 107 (24.3%) | 67 (34.0%) | 29 (47.5%) | *<*0.001 | 698 |
| Furosemide | 24 (4.5%) | 10 (2.9%) | 5 (3.4%) | 9 (22.0%) | *<*0.001 | 531 |
| Spironolactone | 15 (2.8%) | 10 (2.9%) | 3 (2.1%) | 2 (4.9%) | 0.530 | 531 |
| Other diuretics | 271 (38.8%) | 154 (35.0%) | 90 (45.7%) | 27 (44.3%) | 0.025 | 698 |
| Other antihypertensives | 108 (15.5%) | 59 (13.4%) | 32 (16.2%) | 17 (27.9%) | 0.013 | 698 |
| Dyslipidemia | 465 (66.7%) | 274 (62.4%) | 146 (74.1%) | 45 (73.8%) | 0.007 | 697 |
| Total cholesterol (mmol/L) | 4.2 ±1.1 | 4.1 ±1.1 | 4.2 ±1.3 | 4.3 ±1.2 | 0.443 | 690 |
| HDL cholesterol (mmol/L) | 1.2 ±0.3 | 1.2 ±0.4 | 1.1 ±0.3 | 1.2 ±0.4 | <0.001 | 693 |
| Triglycerides (mmol/L) | 1.7 ±1.1 | 1.6 ±1.0 | 2.0 ±1.3 | 1.9 ±1.2 | <0.001 | 693 |
| LDL cholesterol (mmol/L) | 2.3 ±0.9 | 2.2 ±0.9 | 2.2 ±1.0 | 2.4 ±1.0 | 0.708 | 685 |
| Dyslipidemia-related treatment: |  |  |  |  |  |  |
| Statin | 437 (62.6%) | 266 (60.5%) | 132 (67.0%) | 39 (63.9%) | 0.280 | 698 |
| Fibrate | 23 (3.3%) | 11 (2.5%) | 8 (4.1%) | 4 (6.6%) | 0.147 | 698 |
| Ezetimibe | 31 (4.4%) | 19 (4.3%) | 10 (5.1%) | 2 (3.3%) | 0.843 | 698 |
| Current smoking | 103 (14.8%) | 61 (13.9%) | 37 (19.0%) | 5 (8.2%) | 0.080 | 694 |
| Sleep apnea syndrome | 88 (16.8%) | 55 (16.1%) | 24 (16.8%) | 9 (22.0%) | 0.641 | 525 |
| C-reactive protein (mg/L) | 6.5 ±19.4 | 5.5 ±14.6 | 9.0 ±29.0 | 5.9 ±8.8 | 0.294 | 675 |
| Epicardial adipose tissue (cm^3^) | 98.0 ±38.4 | 94.7 ±36.9 | 106.0 ±40.2 | 95.7 ±39.8 | 0.004 | 700 |
| Coronary artery calcium score (AU) | 193.4 ±438.6 | 141.8 ±317.3 | 228.0 ±395.2 | 456.7 ±946.4 | <0.001 | 699 |
| Coronary artery calcium score > 100 AU | 239 (34.3%) | 121 (27.6%) | 92 (46.9%) | 26 (42.6%) | <0.001 | 696 |

*Data are presented as the mean ± standard deviation or n (%). ACE: Angiotensin-converting-enzyme, ARB: angiotensin II receptor blocker, AU: Agatston unit.*

*p-value: comparison between the three classes*

Supplementary Table 4: Association between epicardial adipose tissue volume and nephropathy and components in multivariable analyses

| **Adjustment for** | **Outcomes** | **Odds ratio**  **(by 10 cm^3^ of EAT)** | **[95% CI]** | **p-value** |
| --- | --- | --- | --- | --- |
| - Age (per year) - Gender (Ref: Female) - Ethnicity (Ref: Caucasian) - Diabetes duration (per year) - HbA1c level (per %) - Hypertension - Dyslipidaemia - Body mass index (kg/m²) - GLP1-RA - SGLT2i - Diuretic use - ACE inhibitor or ARB - C-reactive protein (mg/L) | Nephropathy | 1.058 | [1.007–1.112] | **0.027** |
|  | eGFR < 60 mL/min/1.73m² | 1.053 | [0.981–1.131] | 0.153 |
|  | ACR ≥ 3 mg/mmol | 1.059 | [1.007–1.113] | **0.026** |

*ACR: albuminuria/creatininuria ratio, ACE: Angiotensin-converting enzyme, ARB: angiotensin II receptor blocker, CI: confidence interval, EAT: epicardial adipose tissue; eGFR: estimated glomerular filtration rate, GLP1-RA: Glucagon-like peptide-1 receptor agonist, SGLT2i: Sodium/Glucose cotransporter 2 inhibitor*

Supplementary Table 5: Sensitivity analysis. Multivariable analysis considering the same parameters as in table 2 and source study as a fixed effect (7; 8; 13).

*OR: Odds Ratio; CI: Confidence Interval; ACR: Albuminuria/Creatininuria Ratio; eGFR: estimated Glomerular Filtration Rate*

Supplementary Table 6: Sensitivity analysis. Multivariable analysis considering the same parameters as in table 2 with multiple imputation or complete case.

A) EAT volume — Imputation vs Complete-case — Nephropathy

| **Method** | **OR** | **95% CI** | **p-value** |
| --- | --- | --- | --- |
| Multiple imputation | 1.058 | 1.007–1.112 | 0.026 |
| Complete-case analysis | 1.046 | 0.993–1.104 | 0.094 |

B) EAT volume — Imputation vs Complete-case — eGFR < 60 mL/min/1.73m^2^

| **Method** | **OR** | **95% CI** | **p-value** |
| --- | --- | --- | --- |
| Multiple imputation | 1.047 | 0.975–1.123 | 0.204 |
| Complete-case analysis | 1.040 | 0.962–1.124 | 0.324 |

C) EAT volume — Imputation vs Complete-case — ACR ≥ 3 mg/mmol

| **Method** | **OR** | **95% CI** | **p-value** |
| --- | --- | --- | --- |
| Multiple imputation | 1.059 | 1.008–1.114 | 0.024 |
| Complete-case analysis | 1.054 | 0.999–1.112 | 0.053 |

*OR: Odds Ratio; CI: Confidence Interval; ACR: Albuminuria/Creatininuria Ratio; eGFR: estimated Glomerular Filtration Rate*
